# The Genetic Basis of Delayed Puberty

**DOI:** 10.3389/fendo.2019.00423

**Published:** 2019-06-26

**Authors:** Sasha R. Howard

**Affiliations:** Centre for Endocrinology, William Harvey Research Institute, Barts and the London School of Medicine and Dentistry, Queen Mary, University of London, London, United Kingdom

**Keywords:** puberty, constitutional delay of growth and puberty (CDGP), endocrine genetics, self-limited delayed puberty, EAP1, IGSF10, HS6ST1, FTO

## Abstract

Delayed pubertal onset has many etiologies, but on average two-thirds of patients presenting with late puberty have self-limited (or constitutional) delayed puberty. Self-limited delayed puberty often has a strong familial basis. Segregation analyses from previous studies show complex models of inheritance, most commonly autosomal dominant, but also including autosomal recessive, bilineal, and X-linked. Sporadic cases are also observed. Despite this, the neuroendocrine mechanisms and genetic regulation remain unclear in the majority of patients with self-limited delayed puberty. Only rarely have mutations in genes known to cause aberrations of the hypothalamic-pituitary-gonadal axis been identified in cases of delayed puberty, and the majority of these are in relatives of patients with congenital hypogonadotropic hypogonadism (CHH), for example in the *FGFR1* and *GNRHR* genes. Using next generation sequencing in a large family with isolated self-limited delayed puberty, a pathogenic mutation in the CHH gene *HS6ST1* was found as the likely cause for this phenotype. Additionally, a study comparing the frequency of mutations in genes that cause GnRH deficiency between probands with CHH and probands with isolated self-limited delayed puberty identified that a significantly higher proportion of mutations with a greater degree of oligogenicity were seen in the CHH group. Mutations in the gene *IGSF10* have been implicated in the pathogenesis of familial late puberty in a large Finnish cohort. *IGSF10* disruption represents a fetal origin of delayed puberty, with dysregulation of GnRH neuronal migration during embryonic development presenting for the first time in adolescence as late puberty. Some patients with self-limited delayed puberty have distinct constitutional features of growth and puberty. Deleterious variants in *FTO* have been found in families with delayed puberty with extremely low BMI and maturational delay in growth in early childhood. Recent exciting evidence highlights the importance of epigenetic up-regulation of GnRH transcription by a network of miRNAs and transcription factors, including *EAP1*, during puberty. Whilst a fascinating heterogeneity of genetic defects have been shown to result in delayed and disordered puberty, and many are yet to be discovered, genetic testing may become a realistic diagnostic tool for the differentiation of conditions of delayed puberty.

## Introduction

The timing of puberty in humans and other mammals is strongly influenced by genetic regulation. Studies using epidemiological and intra-familial tools give an estimate of 50–80% of the variation in timing of pubertal onset being under genetic control (1, 2). Another illustration of this is the high correlation of the timing of sexual maturation observed between twins (3). Although the precise age of onset of puberty varies within and between different populations, it is a highly heritable phenotypic feature (4). Despite this strong genetic component, there is much that we still do not understand about the physiological control of the timing of onset of, or progression through, puberty (5).

The clinical phenotype of delayed puberty can be a feature of several different conditions (6). However, the most common presentation is with isolated and self-limited delayed puberty (also known as constitutional delay of growth and puberty, or CDGP). Self-limited delayed puberty has been shown in several observational studies to be the commonest cause of delayed puberty in males and females (7). More than 80% of boys and around one-third of girls presenting with late pubertal onset have this disorder of pubertal timing. The term “self-limited” has been coined as in these patients puberty will have commenced by the age of 18 years. Notably, constitutional features involving short stature or slow growth in early childhood are not seen in all patients with “simple” delayed puberty. In a patient presenting with delayed puberty in adolescence there are three main differential diagnoses: (1) central hypogonadism which is functional or temporary, where inhibition of the hypothalamic-pituitary-gonadal (HPG) axis is secondary to chronic disease (in one-fifth of those with late pubertal onset), under-nutrition, excessive exercise, or psychological distress; (2) permanent (central) hypogonadotropic hypogonadism, either congenital hypogonadotropic hypogonadism (CHH) or acquired, with classically low or normal LH and FSH levels (seen in 9% of males and up to one-fifth% of females); and (3) primary hypogonadism, with elevated gonadotropin levels secondary to gonadal failure, low sex steroid concentrations, and failure of negative feedback (in ~7% of males and one-quarter of females with late pubertal onset) (8).

**Figure 1:**
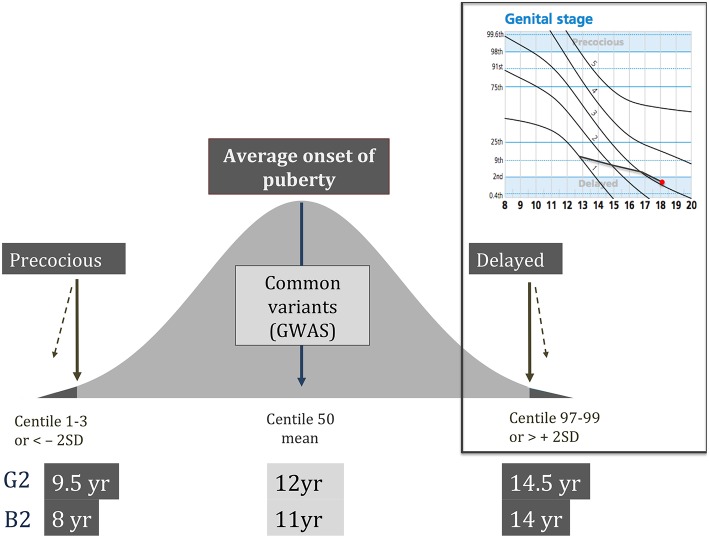
Schematic showing the normal distribution of timing of pubertal onset in the general population, with definitions of precocious and delayed being < or > 2 standard deviations from the mean age, respectively. Top right panel shows an example of a male puberty normogram demonstrating arrested puberty at G3. G–genital stage (Tanner); B–breast stage (Tanner); GWAS–genome wide association studies; SD–standard deviation.

Self-limited delayed puberty represents a timing of puberty onset at the extreme end of normal. Thus, those patients with this condition have a lack of testicular enlargement in males or breast development in females at an age that is 2 to 2.5 standard deviations (SD) later than the population mean (Figure 1) (6). Moreover, children with slow or stuttering progression through puberty, as diagnosed through the use of puberty normograms, can also fall within this diagnostic category (9) (Figure 1). Delay of pubertal development has now been recognized to be associated with several long-term sequelae and is no longer seen as a benign developmental variant (10). These adverse consequences include a higher risk for early natural menopause and poor overall health (11) and negatively affected psychosocial well-being and peer relationships (12). There is some evidence that delayed puberty is associated with lower bone density (13). Adult height can be affected by late pubertal timing but on average it is only slightly below the genetic target (12).

**Figure 2:**
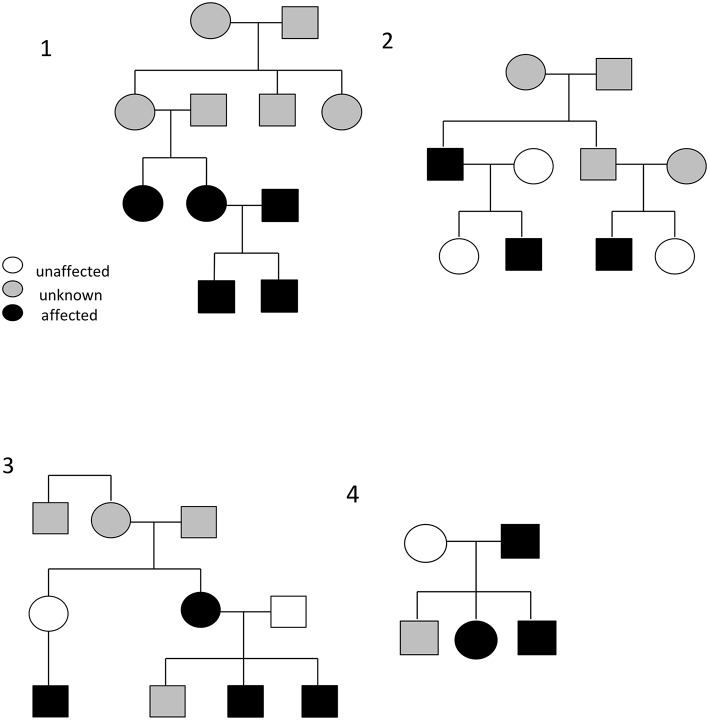
Example pedigrees demonstrating the typical autosomal dominance inheritance pattern seen in self-limited delayed puberty (pedigrees 2 and 4), including bilineal inheritance (shown in pedigree 1), and incomplete penetrance (pedigree 3). Black circles/squares–delayed puberty; clear circles/squares–normal timing of puberty; gray circles/squares–timing of puberty not known.

Between half and two-thirds of those patients with self-limited delayed puberty have a family history of late puberty (14). Observational studies have demonstrated that self-limited delayed puberty is inherited with several different inheritance patterns including autosomal dominant or recessive, bilineal (both parents affected by delayed puberty), and X-linked. Sporadic cases are also observed (Figure 2). However, the majority of families display an autosomal dominant pattern of inheritance (with or without complete penetrance) (14–16). Whilst previously considered to be more common in males, evidence suggests that self-limited delayed puberty is not sex-specific, as within families there are near equal ratios of males and females affected with the trait (16). Indeed, in a cohort review by Winter et al., there were a higher number of female than male relatives affected with delayed puberty (47 females vs. 34 males) (17). The higher number of males that present to a medical team may well be a consequence of referral bias.

The etiology is unknown in the majority of patients with delayed puberty (18, 19). Identification of causal genetic defects in familial delayed puberty is complex for a numbers of reasons. Firstly, delayed puberty is not a rare condition, occurring (by statistical definition) in ~2% of the population. Secondly, whilst some pedigrees display clear Mendelian inheritance patterns it is likely that patients may have a di- or oligogenic (where variants in more two genes contribute to the phenotype) genetic basis for their phenotype in many cases. Thirdly, as noted above, self-limited delayed puberty represents a timing of puberty onset at the extreme end of a near-normally distributed trait in the general population, so there may be a low level of causal variants for this condition seen in population databases. Therefore, we cannot, as is often applied for rare diseases, filter out all non-novel variants from our sequencing datasets when searching for causal variants. Instead, we need to compare the prevalence of all rare and predicted damaging variants in a certain gene between cases and controls, in order to identify those genes that are enriched for deleterious variants in patients compared to the general population (20). Finally, the impact of environmental factors such as nutrition and endocrine disruptors superimposed on genetic regulation can “muddy the waters” for those attempting to isolate definitive genetic causes of delayed puberty.

## Overlap With Common Genetic Variants of Pubertal Timing

### Leptin and Its Pathways

**Figure 3:**
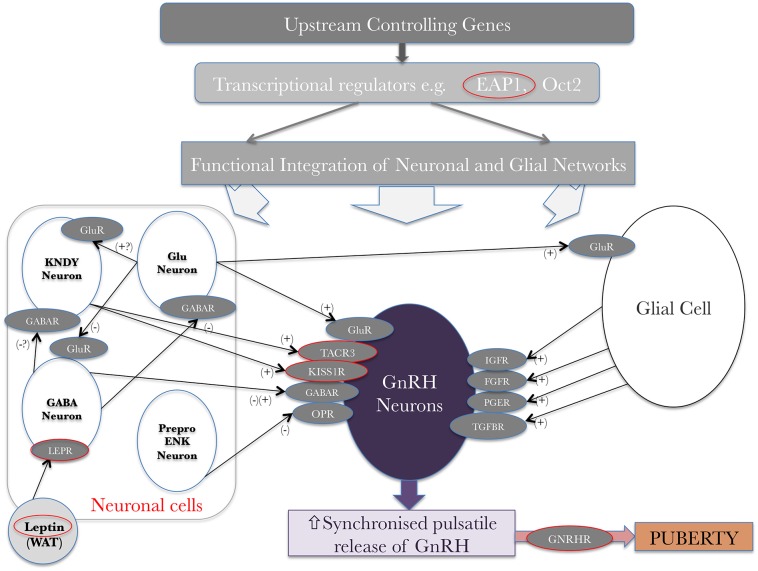
Genetic regulators in the trans-synaptic and glial control of GnRH neurons at the onset of puberty, original idea from Ojeda et al. (24) and adapted from Howard (25) under the CC-BY license. + represents an activating signal, – represents a repressing signal. Red circles highlight genes, mutations in which have been shown to affect pubertal timing. WAT–white adipose tissue; glu–glutamate; gluR–glutamate receptor; KNDY–see text.

The noted secular trend toward an earlier age of pubertal onset in the developed world has been a subject of study for some time. The importance of energy balance and over- or under-nutrition is clear; a minimum level of energy availability is needed for puberty to ensue, but in contrast higher BMI is associated with earlier puberty (21). This latter statement has been seen especially in females (22, 23), but the underlying patho-biology is still not entirely clear. Leptin, a key metabolic hormone and modulator of BMI in humans, is produced from white adipose tissue (Figure 3). It is a major signal of energy sufficiency and mediates, at least in part, the influence of fat mass on pubertal timing. Leptin is a permissive signal for puberty and is necessary for normal reproduction. In females, serum leptin concentrations rise at the onset of puberty (26). Both humans and mice which lack leptin (Lep ob/ob) or its receptor (LepR db/db) show failure to complete puberty and are infertile (27). However, leptin is not the key coordinator in the up-regulation of GnRH signaling pathways at pubertal onset. Leptin alone does not stimulate pubertal onset and, whilst in females leptin concentrations rise during puberty, levels are lower in males and decrease during puberty (28). GnRH neurons do not express LepR therefore leptin cannot act directly to regulate GnRH neurons. Instead its acts indirectly via leptin-sensitive afferents which project to GnRH neurons (29). These afferents are likely to include LEPR-expressing GABA neurons from the arcuate nucleus, nitric oxide (which is required for its action) pathways, mTOR signaling, as well as kisspeptin/neuropeptide Y neurons (30, 31).

### Genome-Wide Association Studies

A key strategy in the attempt to uncover the key genetic regulators of pubertal timing in the general population has been genome-wide association studies (GWAS) of age at menarche and voice-breaking in healthy women and men, respectively. The first locus to be identified as associated with pubertal timing was the single nucleotide polymorphism (SNP) rs314276 in the gene *LIN28B* (32). The major allele of this SNP correlates with earlier breast development and menarche in girls (32). *LIN28B* is a human ortholog of a Caenorhabditis elegans gene important for developmental timing. The lin-28 family regulates, and is regulated by, the let-7 family of microRNAs (miRNAs). However, no human mutations in *LIN28B* have been identified, neither with delayed (33) nor with early puberty (34).

Since this initial discovery several increasingly large meta-analyses have been carried out on GWAS of timing of puberty. Whilst the first of these identified 42 (30 new, 2 previously confirmed and 10 possible) loci for age at menarche (35), an analysis of 182,416 European women encompassing 57 studies (36) identified 106 genomic loci. These meta-analyses are ongoing, but the largest to date which comprises 1000 Genomes Project-imputed genotype data in ~370,000 women has isolated 389 independent signals (*P* < 5 × 10^−8^) for age at menarche (37). The effect size of each of these alleles on the timing of menarche is between 1 week and 5 months. In total the loci identified in this study can explain ~7.4% of the variation in the timing of menarche in the general population, which corresponds to ~25% of the estimated heritability. Together this data suggests that individually many of these genetic variants have a low impact in the general population (37). Hence these huge studies suggest that there is a large degree of heterogeneity in the genetic determinants of normal pubertal timing. A large number of these signals show a significant association with Tanner staging in men and women, implying that the data is applicable to both genders. Additionally, many of these signals have been shown to have concordant effects on the age at voice breaking. However, in women the signals identified have stronger effects on early than on late age of menarche, but in contrast have larger effect estimates for relatively late than relatively early voice breaking in males (37).

Multiple signals in or near genes regulating the HPG axis function have been found by these studies including *LEPR-LEPROT, GNRH1*, and *TACR3*, mutations in which have been shown to be causal in CHH (38, 39). Loci in or near several further genes related to development of the pituitary and its function were also seen, including *POU1F1, TENM2*, and *LGR4*, the last of which acts as an enhancer for the pituitary development factor *SOX2*.

### Energy Metabolism Genes Found by GWAS

In addition to leptin signaling, several other genes implicated in body mass index including *FTO, SEC16B, TMEM18*, and *NEGR1* have been implicated by GWAS as having a role in the timing of puberty. *FTO* had already been identified via GWAS of susceptibility to obesity, and it remains the original and most impactful locus with respect to effect on BMI and risk of obesity (40). Subsequently, using next generation sequencing techniques rare heterozygous variants in *FTO* have been identified in pedigrees with self-limited delayed puberty associated with extreme low BMI and maturational delay in growth in early childhood (41). In a parallel murine experiment, mice that were heterozygous for *FTO* gene knockout were shown to have significantly delayed timing of puberty (41).

A further gene, *IRX3*, identified at the same GWAS locus as *FTO*, was later found also to be of importance in influencing BMI (42); however the evidence from animal models on the effect of FTO on food intake regulation remains robust (43), although its actions may be complex (44). *FTO*-knockout mice (45) and *in vitro* studies have demonstrated that essential amino acids act to modulate the expression of *FTO* and that *FTO* acts downstream to influence mTORC1 signaling (46). mTOR acts as a coupler of energy balance and the activity of the reproductive axis by regulation of the hypothalamic expression of the kisspeptin gene (47). Blockade of mTOR in a rodent model led to delayed vaginal opening with blunting of the positive effects of leptin on puberty onset in food-restricted females (48). However, it is still unknown if the effect of *FTO* on pubertal timing is facilitated via effects on BMI, via mTOR signaling, or by both.

### Other Energy Metabolism Genes

Neuropeptide Y (NPY) is another protein implicated in the regulation of food intake and satiety, as well as the hypothalamic-pituitary axis. NPY increases the response of pituitary gonadotrope cells to GnRH (49), both by stimulating GnRH binding to pituitary GnRH receptors and by its action upstream at the median eminence to potentiate GnRH secretion from GnRH axon terminals (50). Studies with primate models imply that NPY may contribute to the brake that restrains the onset of puberty between infancy and mid-childhood (51). The link between energy homeostasis and reproductive development may also be mediated by ghrelin and other gut-derived peptides (52–54). α-MSH signaling via MC3/4 receptors, acting to increase *Kiss1* expression and mediate the permissive effects of leptin on puberty, has also been implicated recently as an important element in the metabolic control of puberty (55). Lastly, mice lacking the insulin receptor in astrocytes have delayed puberty and irregular estrus cycles, with reduced astrocyte prostaglandin E synthase 2 levels (56). However, roles for the majority of these genes involved in fat mass and metabolic regulation have not been demonstrably shown in human delay of puberty. A small cohort of 31 patients was analyzed for mutations in the ghrelin receptor, or *GHSR*, and 5 patients were found to have point mutations in this gene (57).

## Importance of GnRH Neuroendocrine Network in the Pathogenesis of Delayed Puberty

### Overlap Between GnRH Deficiency and Delayed Puberty

It is biologically very plausible that the pathophysiology of delayed puberty and conditions of GnRH and gonadotropin deficiency share a common genetic basis. Therefore, investigations have been carried out into the role of genes known to cause CHH in the phenotype of isolated delayed puberty. Previous studies in CHH cohorts have found mutations in *HS6ST1, FGFR1*, and recently in *KLB*, not only in small numbers of patients with CHH but also in their relatives with delayed puberty (58–60). Last year, a study was completed that aimed to compare the frequency with which mutations in genes (*n* = 24) known to cause GnRH or gonadotropin deficiency were found in patients with CHH and individuals with self-limited delayed puberty. This comparison found a significantly higher proportion of mutations in the CHH group (51% of CHH probands vs. 7% of delayed puberty probands, *p* = 7.6 × 10^−11^). Whilst this is perhaps unsurprising, a greater degree of oligogenicity in these GnRH deficiency genes was also seen in the CHH group, suggesting a mostly distinct or as yet undiscovered genetic basis of these two conditions (61). Mutations in Kallmann Syndrome (KS) genes such as *ANOS1* and *NSMF*, leading to hypogonadotropic hypogonadism with anosmia, have not been found in individuals with self-limited pubertal delay.

Studies using next generation sequencing to examine cohorts of patients with delayed puberty have identified variants in several CHH genes, particularly *GNRHR, TAC3* and its receptor *TACR3*, but also in *IL17RD* and *SEMA3A* (62). However, these variants have not been tested *in vitro* or *in vivo* for pathogenicity, or investigated for within pedigree segregation. Many syndromic conditions have delayed or absent puberty within the phenotypic spectrum of the condition, see Table 1.

**Table 1:** Genetic syndromes associated with pubertal delay.

**Syndrome**	**Phenotype**	**Genetic defect**
Prader-Willi (63)	Mental retardation, morbid obesity, hypotonia, hypogonadism, growth hormone deficiency, hypothyroidism	Deletions within the paternally imprinted 15q 11.2-12 region
Bardet-Biedl (64)	Mental retardation, obesity, retinitis pigmentosa, post-axial polydactyly, delayed puberty, and hypogonadism	BBS 1-11 (multiple loci) 20p12, 16q21, 15q22.3-23, 14q32.1
CHARGE anomaly (65)	Coloboma, heart malformations, choanal atresia, growth retardation, genital anomalies and ear anomalies, hypogonadotropic hypogonadism, olfactory bulb aplasia, or hypoplasia	*CHD7*
Adrenohypoplasia Congenita (66)	Primary adrenal deficiency and hypogonadotropic hypogonadism	*NR0B1*
Septo-optic dysplasia (67)	Small, dysplastic pale optic discs, pendular nystagmus, Midline hypothalamic defect with DI, single or multiple pituitary hormone deficiency, absent septum pellucidum	*HESX1*
Solitary median maxillary incisor syndrome (68)	Prominent midpalatal ridge, holoprosencephaly, pituitary defects	*SHH*
Borjeson-Forssman-Lehmann syndrome (69)	Mental retardation, gynaecomastia, moderate short stature, truncal obesity	*PHF6*
Hartsfield (70)	Holoprosencephaly, ectrodactyly/split hand and foot malformations, cleft lip and palate, hypogonadotropic hypogonadism	*FGFR1*
Gordon Holmes (71)	Cerebellar ataxia, dementia, chorioretinopathy, anterior hypopituitarism	*RNF216/OTUD4 PNPLA6*

### Heparin Sulfate 6O Sulphotransferase 1

Recently, using whole and targeted exome analysis a mutation in *HS6ST1* was found in one extended pedigree from a large cohort of patients with isolated familial delayed puberty, for the first time without associated CHH in patient relatives (72). All of the six family members in three generations that carried the mutation had a classical self-limited delayed puberty phenotype, with no individuals displaying CHH. A spontaneous onset of puberty was seen in the proband at 14.3 years. A mouse heterozygous knockout model was also examined in parallel. This work substantiated that loss of one allele of *Hs6st1* can provoke pubertal delay but with normal adult reproductive capacity. The *Hs6st1*^+/−^ mice displayed no compromise in their fertility, GnRH neuron or testes development or spermatogenesis and were born at normal Mendelian ratios. However, female mice were seen to have a significant delay in the timing of vaginal opening, a surrogate for onset of puberty in female rodents.

Notably the *Hs6st1*^+/−^ mice had no defects of olfactory bulb morphology and no significant reduction in the total number of GnRH neurons in the hypothalamus or extending to the median eminence to explain the pubertal delay. Instead, this might be mediated by changes in either GnRH neuron activity or other relevant downstream pathways, implied by the expression of *Hs6st1* mRNA in both the arcuate nucleus and paraventricular nucleus (73, 74). These results indicate whilst, as above, many patients with familial self-limited delayed puberty do not carry mutations in CHH genes, perturbations in a single allele of a particular subset of genes that modulate the HPG axis may be enough to result in a phenotype of self-limited pubertal delay. In contrast, more deleterious alterations in these genes, mutations in both alleles of a gene or a heterozygous mutation in combination with mutations in further genes, are needed to produce the more severe phenotypes of CHH and KS (75).

### Immunoglobulin Superfamily Member 10

**Figure 4:**
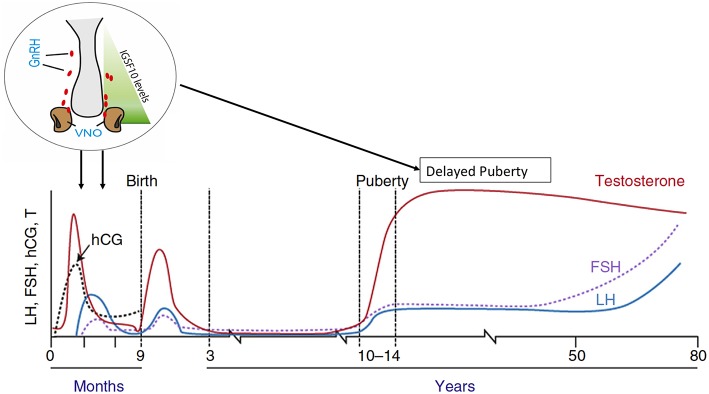
Schematic of the mechanism by which *IGSF10* mutations lead to delayed puberty. Reduced levels of *IGSF10* expression during embryogenesis in the corridor of nasal mesenchyme from the vomeronasal organ to the olfactory bulbs result in delayed migration of GnRH neurons to the hypothalamus. This leads to a phenotype of delayed puberty first evident in adolescence, due to abnormalities of the GnRH neuroendocrine network. Adapted from doi: 10.1210/er.2018-00248.

A further study utilizing whole and targeted exome sequencing methods in the same large Finnish cohort of individuals with familial self-limited delayed puberty, identified deleterious mutations in the *IGSF10* gene in six unrelated families (76). Mutations in this gene affect the migration of GnRH neurons from the vomeronasal organ in the nose to the forebrain during embryonic development (Figure 4). The patients with these mutations presented in adolescence with pubertal delay without features of constitutional delay in growth. Given that a functional GnRH neurosecretory network is required for the onset of puberty, the hypothesis produced from this work is that disruption of GnRH neuronal migration, as caused by aberrant IGSF10 signaling, could result in arrival of fewer (or delayed) GnRH neurons at the hypothalamus. This would then in turn lead to a functional defect in the GnRH neuroendocrine network and an increased “threshold” for the onset of puberty, with a resultant delay. In addition, loss-of-function mutations in *IGSF10* were found in patients with a hypothalamic amenorrhea-like phenotype, implying a shared genetic basis of functional central hypogonadism with both CHH (77) and delayed puberty. However, although deleterious mutations were enriched in CHH patients, there was lack of complete segregation with trait in these permanent hypogonadotropic hypogonadism families, suggesting that haploinsufficiency of *IGSF10* is not sufficient to cause this phenotype. Interesting, mutations in *IGSF10* have very recently also been found in patients with both premature ovarian insufficiency and disorders of neuronal development, and in the same report in a further pedigree with a Kallmann-like phenotype (78). The results of the studies on *HS6ST1* and *IGSF10* in delayed puberty point to a mechanism by which developmental defects in the GnRH system during fetal life can modulate the timing of pubertal onset in adolescence, seemingly without other phenotypic features. It remains to be determined whether these patients have any deficiency of the their long-term reproductive capacity or sexual lifespan.

### Genes Downstream of GnRH

Autosomal recessive CHH is most frequently caused by loss-of-function mutations within the GnRH receptor, accounting for 16–40% of this patient group. Mutations have been found within the extracellular, transmembrane, and intracellular domains of the receptor leading to impaired GnRH action (79). Sequencing studies that have analyzed the *GNRHR* gene in cohorts with self-limited delayed puberty (79), have found just a handful of deleterious mutations. A homozygous partial loss-of-function mutation in *GNRHR* was found in two brothers, one with self-limited delayed puberty and one with CHH (80), and a further heterozygous mutation found in one male with self-limited delayed puberty (81). Far more rarely defects of the glycoprotein hormones luteinizing hormone (LH) or follicle-stimulating hormone (FSH) can lead to CHH, in particular via mutations in the specific β-subunits (82, 83). In women, loss-of-function mutations of *LH*β result in normal or late timing of menarche (following a normally timed onset of puberty) but with later infertility resulting from lack of ovulation (84). In men, similar defects lead to a presentation with absent pubertal development secondary to Leydig cell hypoplasia resulting in testosterone deficiency and failure of spermatogenesis. Women with inactivating *FSH*β mutations display pubertal arrest and primary amenorrhea whilst men have a similar pattern of spontaneous entry into puberty followed by arrest with azoospermia (85). Defects in these two genes do not usually present with a classical picture of self-limited delayed puberty.

Overall, from the evidence we have from current published work we can conclude that although there are some shared gene defects, the genetic basis of CHH and delayed puberty is likely to be due to different, currently unrecognized, genes in many cases (Table 2) (81).

**Table 2:** Non-syndromic genetic defects associated with pubertal delay.

**Phenotype**	**Gene**
Self-Limited Delayed Puberty, Hypogonadotropic Hypogonadism	*HS6ST1* (72) *TAC3* (62), *TACR3* (62), *IL17RD* (62), *GNRHR* (81) *SEMA3A* (62)
Self-Limited Delayed Puberty, Hypothalamic amenorrhea	*IGSF10* (76)
Self-Limited Delayed Puberty	*EAP1* (86)
Constitutional Delay in Growth and Puberty	*FTO* (41)

However, there is a wide spectrum of phenotypes in patients with central hypogonadism, ranging from complete hypogonadotropic hypogonadism, with failure of pubertal development, to partial hypogonadism with an arrest of pubertal development, and even reversible hypogonadotropic hypogonadism in some patients post treatment (87–90). It may be a prudent strategy for clinicians to focus the use of genetic testing of known CHH genes in delayed puberty patients on those patients with either extreme delayed puberty, clear familial inheritance or red flags (such as micropenis, cryptorchidism, anosmia, cleft lip or palate or renal agenesis) which would point to a syndromic or CHH phenotype.

## Transcriptional and Epigenetic Control of GnRH Signaling

### The GnRH Pulse Generator

The central control of pubertal onset, after the mid-childhood period of HPG axis quiescence, is orchestrated by a resurgence of the GnRH pulse generator, with a reduction in central inhibition and a sharp upregulation in the activity of this axis. This activity is permitted by a change in the balance of GABA-glutamate signaling in the brain (91). Around this time morphological changes in GnRH neurons have been observed including increases in dendritic spine density and a simplification of their dendritic architecture. The intensification of kisspeptin signaling in the hypothalamus, one of the key hormonal players in puberty onset, at this time is a consequence of both an increase of kisspeptin synthesis and a rise in the responsiveness of GnRH neurons to kisspeptin stimulation. This mechanism has been well observed in murine models, but also in primates and is relatively conserved during evolution (92). However, what is far less well understood are what the triggers are for this upregulation of kisspeptin biosynthesis in the hypothalamus at the end of the juvenile period. Thus, whilst there is strong evidence that the secretion of kisspeptins from KNDy neurons in the arcuate nucleus is one of the vital stimulatory inputs on the GnRH pulse generator, it is not likely to be the ultimate controller of the release of the puberty brake. Rather, kisspeptin is the conductor of the orchestra of upstream stimulators and repressors influencing the system at this crucial developmental stage (51).

### Transcriptional Control of the GnRH Network

It is likely, therefore, that there is no one single gene that is capable of the hypothalamic control of puberty onset. Instead, we can imagine a hierarchical network of genes acting together to lift the brake applied during the dormancy of the HPG axis in mid-childhood (Figure 3). Data to support this hypothesis have largely come from a systems biology approach (93) and animal models (51), with little data from human subjects. It is clear that transcriptional repression is fundamentally important to the regulation of gene expression in mammals. Transcriptional repressors containing zinc finger motifs, which recognize specific DNA sequences in regulatory regions of the genome, are particularly appealing candidates to have major roles in this governing network (94). Potential key regulators include *Oct-2, Ttf-1, Yy1*, and *Eap1*. *Oct-2* is a transcriptional regulator of the POU-domain family of homeobox-containing genes. *Oct-2* mRNA is upregulated in the hypothalamus in juvenile rodents; blockage of Oct-2 synthesis delays age at first ovulation and hypothalamic lesions which induce precocious puberty (e.g., hamartomas) activate Oct-2 expression (95). *Ttf-1* is another homeobox gene that enhances GnRH expression (96). *Ttf-1* expression is increased in pubertal rhesus monkeys (97). *Yy1* is a zinc-finger transcription factor with crucial roles in normal development and malignancy (98). *Eap1*, or *Enhanced at puberty 1*, codes for a nuclear transcription factor, characterized by a dual transcriptional activity: it both trans-activates the GnRH promoter, which facilitates GnRH secretion, and inhibits the preproenkephalin promoter, which represses GnRH secretion. *Eap1* mRNA levels increase in the hypothalamus of primates and rodents during puberty, and Eap1 knockdown with siRNA causes delayed puberty and disrupted estrous cyclicity in a rodent model (99–103). Therefore, *Eap1* transcriptional activity facilitates the initiation of female puberty, in a manner that is independent of hypothalamic Kiss1 expression (101). *Eap1* gene expression is itself regulated by both activation by *Ttf-1*, and repression by *Yy1* and a further transcriptional regulator *Cux1* (104).

### Enhanced at Puberty 1

A very recent discovery is of the first human *EAP1* mutations that appear to be causal for self-limited delayed puberty in two families (86). The affected individuals from these two families had classical clinical and biochemical features of self-limited delayed puberty, with presentation at more than 15.5 years with delayed onset of Tanner stage 2 and delayed peak height velocity. Both probands had spontaneous pubertal development by the age of 18 years without testosterone therapy, thus excluding CHH. By whole exome sequencing of probands with familial delayed puberty two highly conserved variants—one in-frame deletion and one rare missense variant in *EAP1*—were identified. Using a luciferase reporter assay, EAP1 mutants showed a reduced ability to trans-activate the GnRH promoter compared to wild-type EAP1, due to reduced protein levels caused by the in-frame deletion and sub-cellular mis-location caused by the missense mutation. This study also demonstrated by chromatin immunoprecipitation that EAP1 binding to the GnRH1 promoter increases in monkey hypothalamus at the onset of puberty.

### Polycomb Complex Genes

Furthermore, evidence from a recent study has emphasized the importance of the transcriptional control of the Kisspeptin gene *Kiss1*. This regulation by the polycomb complex proteins EED and Cbx7 is thought to be an important transcriptional repressive mechanism to prevent the premature onset of puberty (105). In the latter stages of mid-childhood there is an increase in the methylation of the promoters of these genes, resulting in a reduction in expression, as well as a decrease in the binding of EED on the *Kiss1* promoter. This inhibition of *Kiss1* repression also correlates with reduced expression of transcription factors containing certain zinc finger motifs. Moreover, there is also reorganization of the chromatin status and changes in histone methylation to accompany the loss of these polycomb complex proteins from the *Kiss1* promoter (106). Studies on both rats and goats also provide data on changes in histone acetylation and gene methylation resulting in alterations in gene expression during puberty (107, 108).

### Epigenetic Mechanisms in the Timing of Puberty

There are a number of different epigenetic mechanisms that may have importance for the regulation of the pubertal timing, including imprinting. Imprinted genes are known to influence the timing of several key developmental stages in humans including weaning and adrenarche. In general, paternally expressed genes promote later childhood maturation and maternally expressed genes promote a more premature maturation (109). This holds true for two paternally inherited genes, *MKRN3* and *DLK1*, which are associated with age at menarche in girls and voice-breaking in boys from the GWAS discussed above (37). Variants in both of these genes have been discovered in patients with familial central precocious pubertal timing, with paternally-inherited mutations leading to the expression of the phenotype (110, 111). *MKRN3* is thought to contribute to the puberty brake restraining the HPG axis via inhibition of GnRH release. This gene encodes Makorin Ring finger protein 3, a zinc finger protein containing a C3HC4 motif (known as a RING domain) associated with E3 ubiquitin ligase activity (112, 113). Since *MKRN3* expression in the arcuate nucleus falls in murine models between birth and weaning, and in humans serum concentrations decline at puberty onset, it is thought to have an inhibitory effect on the GnRH network (114, 115). This supports the hypothesis that the onset of puberty is a consequence of the removal of gonadotropic axis repression. However, what is still unclear is where *MKRN3* is placed in this hierarchy of gene regulators controlling kisspeptin levels. Very new data has demonstrated that knock-out of *MKRN3* in pluripotent stem cells does not affect *GNRH1*-expression when these cells are later differentiated into neurons (116). In terms of delayed puberty, mutations in neither *MKRN3* nor *DLK1* genes have been described in human patients with these conditions.

### Imprinting and Pubertal Timing

Prader-Willi syndrome (PWS) is frequently caused by disorders of imprinting and is often associated with either absent or delayed puberty (117). In most patients with PWS the syndrome is due to a deletion of a cluster of imprinted genes (including *MKRN3*) on the paternally inherited copy of chromosome 15 (paternal deletion), or by inheritance of both copies of this cluster from the mother (maternal uniparental disomy) (118). Precocious puberty is relatively uncommon in PWS (119), but most individuals show some degree of pubertal failure, with one or a combination of an absent pubertal growth spurt, hypogonadotropic hypogonadism, cryptorchidism, underdeveloped genitalia, or primary amenorrhea (120). The probable explanation for the rarity of precocious puberty in individuals with PWS, despite the lack of *MKRN3* expression, is the effects of other genes inactivated by the imprinting defect, in particular *MAGEL2* (121, 122). This points to a complex role for imprinted genes in the pubertal timing, with tissue type and developmental stage specific gene expression (109, 118).

### Non-coding RNAs

Evidence from murine models has demonstrated that non-coding RNAs can act as epigenetic modulators of the timing of puberty. Specific microRNAs play a role in the epigenetic up-regulation of GnRH transcription during what is known in mice as “the critical period,” or infantile mini-puberty in humans (123). A key pair of microRNAs (miR-200 and mIR-155) are thought to regulate Gnrh1 expression, and to control the expression of two important transcriptional repressors of Gnrh1, Zeb-1, and Cebpb. There is an associated increase in the transcriptional activation of GnRH1 with a reduction in Zeb-1 and Cebpb, the latter a nitric oxide-mediated repressor of Gnrh1 that acts both directly and through Zeb1. These changes lead to the up-regulation of *Gnrh1* synthesis in GnRH neurons (123). Moreover, miR-7a2 has been demonstrated to be essential for normal murine pituitary development and HPG function, with deletion in mice leading to hypogonadotropic infertility (124).

### Endocrine-Disrupting Chemicals

The increase of kisspeptin and GnRH expression in the hypothalamus at puberty is therefore the result of the actions of an intricate arrangement of repressing and activating transcription factors controlling *Kiss1* and *GnRH1* transcription, with these being themselves influenced by several epigenetic mechanisms including DNA methylation, histone modification and non-coding RNAs (106, 123, 125, 126). Moreover, these epigenetic mechanisms are possible facilitators of gene-environment interactions that also have influence on the hypothalamic regulation of puberty. A number of different sources of evidence have demonstrated that the brain epigenome at puberty is affected by environmental disturbances (107). Endocrine-disrupting chemicals (EDCs), often found in products commonly used in the developed world, have been considered as a potential cause for pubertal timing disturbance for many years, with increasing concern among the lay population (127). Many and varied substances have been identified as possible EDCs, such as polybrominated biphenyls, bisphenol A, atrazine (herbicides), and glyphosate, but also common medicines including paracetamol and betamethasone (128–131). It has been observed that adolescents who have been exposed to the estrogenic insecticide DTT and then adopted internationally display early or precocious pubertal timing (132).

The most important timing of EDC exposure for impact on pubertal timing was historically considered to be in late childhood, but there is now clear data that there may be prenatal and infantile origin of alterations in the timing of puberty. *In utero* exposure in males to EDCs, in particular to phthalates, can result in under-masculinization of genitalia (133). Moreover, exposure of pregnant rodents to EDCs has been associated with epigenetic alterations in testis as well as other systemic effects. This together suggests that epigenetic changes in the fetal period are a potential mechanism for the hypothalamic effects of prenatal exposure to EDCs (131). These effects may manifest in pregnant rodents, their unborn fetus but also into the next two or more generations as well (134).

However, it is difficult to definitively demonstrate a mechanism of action for EDCs through the premature activation of the hypothalamic GnRH pulse generator. Recently, exposure of female mice to arsenic *in utero* was shown to alter the hypothalamic expression not only of GnRH and LH but also of their upstream transcriptional regulators, in particular Oct-2 and Ttf-1 (135). Mice exposed to arsenic demonstrated precocious puberty with premature vaginal opening, a marker of the onset of puberty rodents. However, in most datasets it has been difficult to unpick the most likely differing, and possibly conflicting, influence of varying doses and combination of EDCs affecting estrogenic, androgenic or other pathways, and changes in effects depending on age and length of exposure (136).

## Future Directions

Over the last 2 years there have been very exciting developments in the understanding of the genetic basis of delayed puberty, particularly with respect to the transcriptional and epigenetic control of the GnRH “master switch.” We anticipate further discoveries in the near future that will help to elucidate these control mechanisms and better understand the genetic predisposition to familial delayed puberty and to conditions of functional hypogonadism. It is, of course, hoped that this knowledge can be rapidly translated into more efficient clinical diagnosis and management.

## Conclusion

Puberty represents the remarkable transition from childhood to adult life with the attainment of reproduction and adult stature. The onset of puberty is elicited by the re-activation of the HPG axis, which is first functional in fetal life, through a rise in the pulsatile release of hypothalamic GnRH. Puberty can be deemed the consequence of a neurodevelopmental program, that begins prenatally but has many features in common with the postnatal development of other neuronal processes. However, its unique feature is as a functional system that lies dormant for most of childhood and then reactivates in the majority of the population within a short time window. This timing is controlled by genetic factors, relies upon an intact hormonal axis and influenced by the environment. It is thus not so surprising that pubertal delay and even aberrant pubertal development are not infrequent human pathologies.

**Figure 5:**
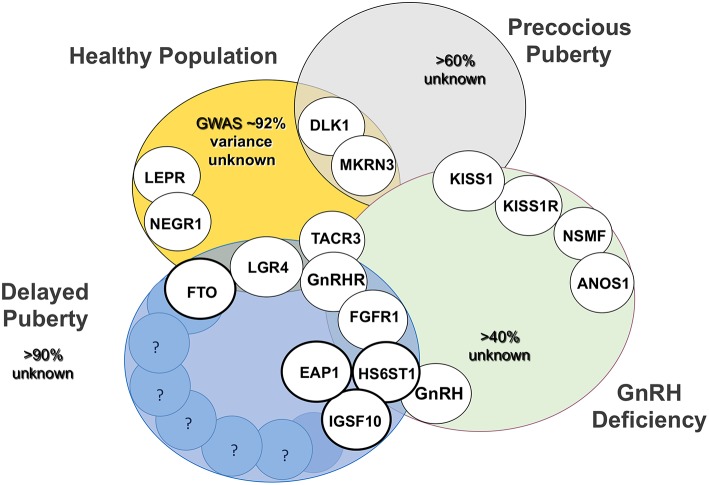
Established genetic basis of common genetic variants of pubertal timing from genome wide association studies (GWAS), conditions of GnRH deficiency (CHH and KS), precocious puberty and delayed puberty and their overlap. Activating and inactivating mutations in KISS1 and KISS1R cause the opposite phenotypes, precocious puberty and CHH, respectively. Bold circles highlight those genes, mutations in which have been identified in familial delayed puberty. Adapted from Howard (25) under the CC-BY license.

The genetic regulators that determine timing of puberty in the general population, a trait that follows a skewed near-normal distribution, have relevance to conditions of delayed and even aberrant pubertal onset (Figure 5). There is also overlap between those pathways found to be defective in self-limited delayed, precocious, and absent puberty conditions, with the phenotype varying dependent on the impact of the gene defect and mutational burden. So whilst there are shared pathogenic mechanisms between these conditions, there is also much heterogeneity in the genetic changes responsible for delayed puberty. Defects in GnRH neuronal development and function, transcriptional regulation of the HPG axis, epigenetic mechanisms including DNA methylation, histone modification and non-coding RNAs, and metabolic and energy homeostatic derangements, can all lead to the final common pathway of delayed puberty. Moreover, these genomic regulators can exert their influence in fetal life, during postnatal development and in mid-childhood, all having an effect in adolescence on pubertal timing.

Genetic testing may allow the translation of this understanding to benefit patient care in the future: as a diagnostic tool for the investigation of delayed puberty, by informing the natural history of the condition, possible inheritance in the individual's family and optimization of treatment. Rapid and accurate diagnostic testing in clinic would greatly improve patient care and most likely represent a significant advantage in terms of health economics.

## Author Contributions

The author confirms being the sole contributor of this work and has approved it for publication.

### Conflict of Interest Statement

The author declares that the research was conducted in the absence of any commercial or financial relationships that could be construed as a potential conflict of interest.
